# Handbook of bioinspired algorithms and applications

**DOI:** 10.1186/1475-925X-5-47

**Published:** 2006-07-31

**Authors:** Max E Valentinuzzi

**Affiliations:** 1 Instituto Superior de Investigaciones Biológicas (INSIBIO), CONICET, Argentina; 2 Universidad Nacional de Tucumán (UNT), Argentina

After more than half a century of fascinating and glaring development, *Bioengineering and Biomedical Engineering *have come so much closer together in a single general definition that few, if any, dare now questioning it: the application of knowledge, principles and techniques of the *Engineering Sciences *to the understanding and solution of problems of the *Biological Sciences*. Boundaries, as once claimed and often discussed in meetings, have faded away.

Many years ago, the term *Bionics *(now forgotten) was coined, meaning the inverse process, that is, application of knowledge taken from the *Biological Sciences *to solve or help in the solution of problems of *Engineering *practice at large, or simply to get ideas based in the former sciences. Traditional early examples frequently mentioned in the literature to illustrate the point were the snakes' infrared sensors and the ultrasound dolphin emissions used, respectively, to locate a prey and for orientation, as animal inspiration for their military similar counterparts.

Well, this dense handbook falls within the latter concept, as anticipated in its title by the word "bioinspired". Thus, it is quite pertinent to directly quote from the Preface: "The *Handbook *seeks to provide an opportunity for researchers to explore the connection between biologically inspired techniques and the development of solutions to problems that arise in a variety of problem domains." Enrique Alba and Carlos Cotta, from Málaga, Spain, ask in Chapter 1, on "Evolutionary Algorithms", if we can learn, and use for our own profit, the lessons taught by Nature. They declare themselves strongly for the affirmative and define as evolutionary algorithms a collection of optimization techniques whose functioning is loosely based on metaphors of biological processes. Clearly, at the very outset, authors state what the potential reader might expect and whom the content is addressed to.

Section I, devoted to Models and Paradigms, takes about one fourth of the book (175 pages out of a total of 663), while Section II deals with Applications and Domains, although the division is not really very much clear-cut as a certain degree of overlapping between the two is easily detected. Somewhat arbitrarily, I selected a few chapters to briefly comment about for they appeared to me as rather appealing. However, it does not mean that the others are not. Besides, a book of this size and scope supersedes a full detailed description.

Chapter 3, on "Ant Colony Optimization", by Michael Guntsch and Jürgen Branke, from Karlsruhe, Germany, call our attention as they describe social insects because such colonies are capable of solving a number of optimization problems that none of the individual insects would be able to solve by itself, as for example finding short paths when foraging for food, task allocation when assigning labor to workers, and clustering when organizing brood chambers, all of which are problems that have counterparts in real world optimization problems. The Travelling Salesman Problem is presented as a classical example.

The so called "Swarm Intelligence" (Chapter 4, by Mohamend Belal, Jafaar Gaber, Hoda El-Sayed and Abdullah Almojel, respectively, from Cairo, Egypt, UTMB (place not specified), France, Bowie, Maryland, USA, and Riyadh, Saudi Arabia) is closely related to the previously mentioned chapter, but these authors include in the word "swarm" mammalian herds, fish shoals and bird flocks. They conclude that swarm intelligence is a rich source of inspiration for our computer systems with features that are specific for distributed computing, including auto-configuration, autonomy, robustness and adaptability, among others. Envisioned areas of application are nanotechnology, massively parallel super-computers and embedded systems.

"Parallel Genetic Programming" is the subject developed in Chapter 5 by Francisco Fernández de Vega, from Mérida, Spain. Such knowledge can be used in Field Programmable Gate Arrays (FPGA), that is, integrated devices to implement digital circuits and, most surprisingly, in burn diagnosing, to predict how a burn will evolve and how to choose the best suitable treatment. The latter constitutes a nice unexpected biomedical engineering example.

Another attractive source is presented in Chapter 8, "Optimization via Gene Expression Algorithms", by Forbes Burkowski, from Waterloo, Canada. Genetics is here the inspirational font based, essentially, in the Central Dogma of Biology, as introduced in the 1950's by Watson and Crick when they stated that information flows from DNA (deoxyribonucleic acid) to RNA (ribonucleic acid) to protein synthesis. For example, the binary representation of a gene is formatted to represent a feasible solution of some optimization problem and a fitness function is applied to the gene to assess how well the feasible solution meets the requirements of the problem. If the gene has produced a high fitness evaluation then the gene stays in a population of such genes, otherwise it is replaced.

Some people may shy away if they read early in Chapter 9 ("Dynamic Updating DNA Computing Algorithms", by Zhiquan Frank Qiu and Mi Lu, from Chandler, Arizona, and College Station, Texas, USA, respectively) that DNA computing can be used to solve problems currently intractable on even the fastest electronic computers and that a strong background is needed in both DNA molecule and computer engineering to just tackle these algorithms ... well ... sorry, but this is the way it is. No doubt, Science and Technology progress by leaps and bounds pushed ahead by eager clear-minded young guys who see the way through, always sitting on the shoulders of those who came first, but often not realizing of this fact. As John Gribbin said in his beautiful book [1]: "There has to be an element of speculation – a guess [made by someone], based on past observations and experiments, combined with intuition about the way the world works".

In its 25 chapters, Section II probes into a wide variety of applications. For example, Chapter 12, entitled "Setting Parameter Values for Parallel Genetic Algorithms: Scheduling Tasks on a Cluster", by Michelle Moore, from Corpus Cristi, Texas, USA, makes use of genetic concepts to several common everyday problems, such as, a shipping company handles containers of different sizes and shapes and wants to pack the maximum possible number into a fix space, and the packing plan must be generated for each truck; or an airport wants to determine the fastest pattern for their fleet of snow plows and dump trucks for clearing the snow from the runways, since heavier snowfall will require more trips, the rate of snow fall must be included in the computations.

Chapter 19, by Borut Robić, Peter Korošec and Jurij Šilc, from Ljubljana, Slovenia, deals with "Ant Colonies and the Mesh-Partitioning Problem", while Horst F. Wedde and Muddassar Farooq, from Dortmund, Germany, in Chapter 21 develop routing algorithms inspired by honey bee behaviour. All this material is quite amazing, indeed, for it brings together vastly different pieces of information such as important organizational principles of a honey bee colony that enabled technologists to develop robust routing algorithms. Suffice it to say that the background knowledge stems in the seminal contribution of the Nobel Prize Karl von Frisch, who in 1944 made a revolutionary discovery about the communication system employed by bees to indicate flower sites around the hive. The colony is able to optimize its stockpiles of nectar, pollen and water through an intelligent allocation of labor among different specialists, which communicate with each other using a sophisticated protocol consisting of signals and cues. The dance and foraging behaviour inspired the development of fault-tolerant, adaptive and robust routing protocols.

Mobile telecommunication systems, training of neural networks, electrical engineering design, biomimetic models for wireless sensor networks are some of the many areas that benefit from biology, which, as perhaps some people might like to think, follows the blueprint of The Great Engineer.

It is a handbook, and as such, it is for consultation, not for studying. The reader must be knowledgeable in the specific subject he/she is looking for. The book abounds in acronyms and technical jargon that makes it hard to read, without counting the mathematics involved in many of its chapters, as for example Chapter 24, "Synthesis of Multiple-Valued Circuits by Neural Networks", by Alioune Ngom and Ivan Stojmenović, from Ontario, Canada. I do not mention this as a disadvantage; on the contrary, it means the content goes deep and to the point and the potential user has to be adequately prepared. References are ample and pertinent offering to the reader further sources to search. Moreover, both editors and all contributors are experts in the subjects they deal with. It is, no doubt, a necessary repository piece of reference material in technical libraries, computer labs and for any group devoted to the development of optimal algorithms.

Finally, since it is difficult for me to resist the temptation, let me quote almost verbatim from [2]: "Saint Mathesis, patron of mathematics, used to be surrounded by seven lamps: *lampas utilitatis, lampas imaginationis, lampas poesis, lampas infinitatis, lampas mysterii, lampas religionis and lampas decoris*. Bioengineering, bionics, bioinspiration ... as it better may suit your own feelings ... strongly supported by mathematics, searches quantification and thus, no doubt, these lamps also illuminate its course because it is certainly useful (*utilitatis*) for it applies directly or indirectly to the human being, it requires tremendous imagination (*imaginationis*) and creativity (*poesis*), it extends unlimited in all directions (*infinitatis*) branching off here and there, while it shines with some degree of mystery (*mysterii*) when penetrating the darkness of the unknown, so spurring curiosity, and shows also even mystical and mythical edges when human, ethical and religious aspects are touched on (*religionis*); finally, and perhaps best of them all, it is beautiful (*decoris*)". This book adds new paths to follow, new practical applications and an enormous amount of imagination. The *Seven Lamps *keep spreading their generous light beams all over.
